# Synthesis of nitrogen doped carbon quantum dots/magnetite nanocomposites for efficient removal of methyl blue dye pollutant from contaminated water†

**DOI:** 10.1039/c8ra00158h

**Published:** 2018-02-23

**Authors:** Aschalew Tadesse, Dharmasoth RamaDevi, Mabrahtu Hagos, GangaRao Battu, K. Basavaiah

**Affiliations:** Department of Inorganic and Analytical Chemistry, Andhra University Visakhapatnam-530003 India klbasu@gmail.com; AU College of Pharmaceutical Sciences, Andhra University Visakhapatnam-530003 India; Department of Applied Chemistry, Adama Science and Technology University Adama-1888 Ethiopia; Faculty of Natural and Computational Sciences, Woldia University Woldia-400 Ethiopia

## Abstract

As a remedy for environmental pollution, a simple synthesis approach has been developed to prepare nitrogen doped carbon quantum dot/magnetite nanocomposites (Fe_3_O_4_@NCQDs NCs) using non-toxic and cost effective lemon juice as precursor for removal of organic dye pollutant. Fe_3_O_4_@NCQDs NCs were characterized by using UV-Vis spectroscopy, FTIR, XRD, FESEM, EDS, TEM, VSM and TGA/DTA. TEM results show spherical shaped Fe_3_O_4_@NCQDs NCs with an average particle size of 5 nm. Batch adsorption studies were done to investigate the tendency of the nanocomposites to remove representative methyl blue (MB) dye from aqueous solution. The effects of MB dye concentration, dosage of Fe_3_O_4_@NCQDs NC adsorbent, pH, contact time and temperature were optimized by varying one variable while all the other parameters were kept constant. The experiment showed rapid removal of MB dye within 20 minutes with an adsorption efficiency of over 90.84% under optimum conditions. The adsorption process fits the Freundlich isotherm model well with *R*^2^ and *n* values of 0.993 and 1.842, respectively, at 298 K indicating the feasibility of the adsorption process. The adsorption process is spontaneous and involves exothermic behaviour as confirmed by thermodynamic studies. From a kinetic study, it was found that the pseudo-second order model is more suitable to describe the adsorption process than the pseudo-first order model for adsorption of MB dye onto Fe_3_O_4_@NCQDs NCs.

## Abstract 2

Herein, we report the green synthesis of magnetic, nitrogen doped carbon quantum dot/Fe_3_O_4_ NPs using aqueous lemon extract for the efficient removal of organic dye pollutants from contaminated water.

## Graphical abstract

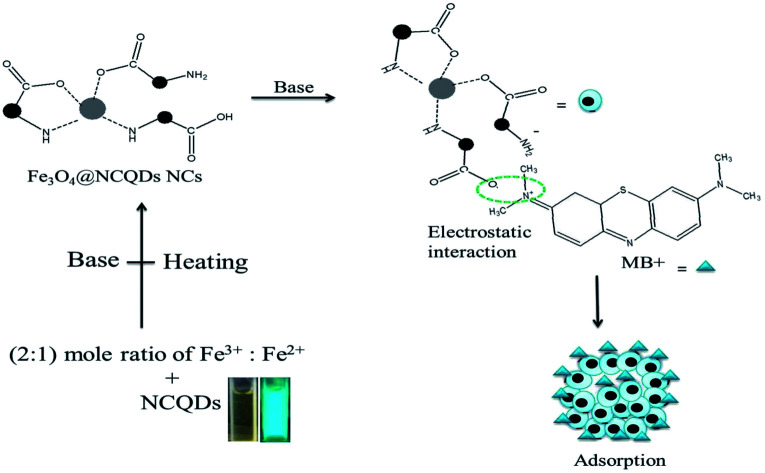

## 1 Introduction

During the past decades, the release of large quantities of toxic, carcinogenic and non-biodegradable organic dye pollutants into aquatic systems has continuously increased due to rapid industrialization, civilization and agricultural activity.^1^ The presence of organic dye pollutants even at trace levels in effluents is very dangerous to human and aquatic life. The organic dye pollutants remain highly visible, resistant to aerobic digestion and stable to oxidizing agents due to their complex chemical structure and synthetic origin.^2^ Therefore, it is highly desirable to develop an environmental friendly, highly efficient and cost effective method for removal of organic dye pollutants from wastewater effluents even at trace levels.

Different treatment methods such as biological treatment,^3^ flocculation–coagulation,^4^ adsorption,^5^ membrane filtration^6^ and oxidation^7^ have been used to remove dyes from wastewater effluents. Among these methods, the adsorption method is the simplest, efficient and low cost method for removal of dyes from wastewater effluents with no generation of byproducts and various natural and synthetic materials have been used as adsorbents.^8,9^ Recently, nanoscience and nanotechnology research introduced nanoadsorbent with more efficient adsorption, low cost and recyclable properties. The most repeatedly investigated nanoadsorbent is magnetite nanoparticles (Fe_3_O_4_ NPs) based nanomaterials due to their excellent magnetic, biocompatible properties, facile synthesis and ease with which they may be tuned and functionalized for specific applications.^10,11^ Moreover, the most important advantage of Fe_3_O_4_ NPs is its easy separation and purification after application by an external magnet due to its magnetic properties.^12,13^ However, bare Fe_3_O_4_ NPs could easily aggregate in aqueous system to reduce the energy associated with surface area to volume ratio and the strong dipole–dipole attraction between particles and easily undergo oxidation and thus limits their technological applications.^14,15^ To effectively use the advantage of Fe_3_O_4_ NPs for technological applications, researchers have been continuously designing the aggregate free Fe_3_O_4_ NPs *via* surface modification and functionalization.^16–18^

A wide range of chemicals such as chitosan, activated carbon, polymers, graphene quantum dots, graphene oxide, multiwall carbon nanotubes, *etc.* have been employed for preparation of Fe_3_O_4_ NPs based nanocomposites for pollution abatement.^19–23^ In preparation of Fe_3_O_4_–chitosan nanocomposites, the surface of magnetite modified with amine and hydroxyl groups on chitosan.^24^ As a class of newly emerging fluorescent nanomaterials, carbon quantum dots (CQDs) have offered tremendous opportunities for a wide scope of applications due to its excellent properties like good stability and solubility in water, low cost and biocompatibility.^25,26^ Tremendous promise has been shown in different applications by compositing carbon quantum dots with nanoparticles.^27^ Similar to chitosan, nitrogen doped carbon quantum dots have reactive amine and hydroxyl groups which are amenable to chemical modifications and therefore, in nanocompositing Fe_3_O_4_ with nitrogen doped carbon quantum dots (NCQDs), amine and carboxyl groups on NCQDs modify the surface of Fe_3_O_4_ and protect the nanocomposites from aggregation. In addition, nitrogen doped carbon quantum dots (NCQDs) can preserve structural stabilization of Fe_3_O_4_ NPs as capping agent and improve the surface of the materials by providing important functional groups which are important for interaction of the nanocomposites with chemical pollutant in environment.^28^

In this paper, we report the design and synthesis of magnetic and eco-friendly Fe_3_O_4_@NCQDs NCs *via* coprecipitation using lemon juice as precursor. The as prepared Fe_3_O_4_@NCQDs NCs have been applied for efficient removal of methyl blue (MB) dye from contaminated water. The effects of various experimental conditions such as contact time, initial concentration, pH, temperature and adsorbent dosages on the removal efficiency of MB were evaluated through a batch adsorption experiments.

## 2 Experimental section

### 2.1 Chemicals and reagents

Iron(iii) chloride hexahydrate (FeCl_3_·6H_2_O) and iron(ii) sulphate heptahydrate (FeSO_4_·7H_2_O) were purchased from Merck, India. Fresh lemon fruits were purchased from the local store nearby Andhra University. 100 mL stainless steel Teflon lined autoclave was used for hydrothermal synthesis of NCQDs. Milli-Q water was used throughout the experiments. Ethylenediamine was purchased from LOBA Chemie, Mumbai, India. All the reagents used are analytical grade and used as received without any further purification.

### 2.2 Synthesis of NCQDs from lemon juice

Hydrothermal method was used to synthesis nitrogen doped carbon quantum dots (NCQDs) by taking 20 mL of lemon juice and 2 mL of ethylenediamine in a 100 mL Teflon-lined stainless steel autoclave and heated at 200 °C in furnace for 3 hours. The obtained black paste was dissolved in 15 mL of water and centrifuged at 3000 rpm for 15 minutes to remove insoluble matter. Dichloromethane was added to the brown solution formed and centrifuged at 3000 rpm for 20 minutes to remove unreacted organic moieties. The aqueous layer was separated from lower organic layer and centrifuged at 12 000 rpm for 20 minutes thrice to remove larger size particles and the brown yellowish solution was finally obtained. To further get the smaller particle size of NCQDs, cleaning was done using column chromatographic separation in help of silica gel and dichloromethane as solvent. The resulting NCQDs was characterized and used for preparation of novel Fe_3_O_4_@NCQDs NCs.

### 2.3 Synthesis of Fe_3_O_4_@NCQDs NCs

Syntheses of Fe_3_O_4_@NCQDs NCs were done *via* coprecipitation reaction. In the procedure, 100 mL aqueous solution of 2 : 1 molar ratio of metal salts Fe^3+^ (1.1127 g FeCl_3_·6H_2_O) and Fe^2+^ (0.5708 g FeSO_4_·7H_2_O) was added in 250 mL three neck round bottom flask and the reaction was carried out for one hour under constant stirring in atmospheric nitrogen at 80 °C. To the reaction flask, 25 mL diluted NCQDs aqueous solution (5 mg mL^−1^ and 10 mg mL^−1^) was added and reaction continued for 30 minutes. Then 20 mL of 2 M NaOH was added drop wise. The reaction was allowed to continue under stirring for 2 hours at 80 °C. Finally, the black precipitate was obtained and separated by decantation with help of external magnet, washed several times with Milli-Q water, and dried under vacuum at room temperature. Bare Fe_3_O_4_ NPs was synthesized in the same procedure without using NCQDs.

### 2.4 Characterization

UV-Vis absorption spectra of the synthesised NCQDs, Fe_3_O_4_ NPs and Fe_3_O_4_@NCQDs NCs were obtained using a UNICAM UV 500(Thermo Electron Corporation). Fourier transform infrared spectra (FTIR) were obtained over the range of 400–4000 cm^−1^ using a SHIMADZU-IR PRESTIGE-2 Spectrometer. X-ray powder diffraction (XRD) pattern were recorded using PANalyticalX'pert pro diffractometer using Cu-Kα1 radiation (45 kV, 1.54056 Å; scan rate of 0.02 degree per s). The morphology and microstructures of the synthesized Fe_3_O_4_@NCQDs NCs were investigated by transmission electron microscopy (TEM) and high resolution transmission electron microscopy (HRTEM, Jeol/JEM 2100, LaB6) operated at 200 kV. Further morphology and composition of Fe_3_O_4_@NCQDs NCs were characterized using field emission scanning electron microscopy (FESEM, Zeiss Ultra-60) equipped with X-ray energy dispersive spectroscopy (EDS). Magnetic property of the material was determined at room temperature using vibrating sample magnetometer (Lakeshore VSM 7410). Composition of the Fe_3_O_4_@NCQDs NCs was further confirmed by thermal analysis using thermogravimetric and differential thermal analysis (TGA/DTA) of Perkin Elmer STA 6000 with TG sensitivity of 0.2 mg and DTA sensitivity of 0.06 mV.

### 2.5 Adsorption studies

To study adsorption efficiency of Fe_3_O_4_@NCQDs NCs for representative methyl blue (MB) dye solution from polluted water, the usual batch adsorption experiments were carried out using a series of conical flask of 100 mL capacity under covered conditions to prevent contamination and removal of dye solution from the flask during stirring. The effects of MB dye concentrations, dosage of Fe_3_O_4_@NCQDs NCs adsorbent, contact time, pH and temperature were optimized by varying one variable while all other parameters kept constant. For isothermal studies, experiments were performed at 293, 298 and 303 K with various initial MB dye concentrations and optimum dosage of adsorbent, contact time and pH. The kinetic experiments were performed at optimum dosage, temperature, pH and dye concentrations at constant time intervals. In the procedure, 100 mL of 10 ppm dye solution was taken in flask and 50 mg of adsorbent added and stirred at 293 K temperature. After certain time (*t*) of adsorption, adsorbent was separated from solution using external magnet and the unadsorbed MB concentration in the solution was determined using a UV-Vis spectrophotometer at *λ*_max_ of 664 nm. The MB uptake and percentage adsorption were calculated using eqn (1) and (2).

1 *Q*_e_ = (*C*_0_ − *C*_e_) × *V*/*m*

2 % Adsorption = ((*C*_0_ − *C*_e_)/*C*_0_) × 100%

where, *C*_0_ and *C*_e_ are the initial and equilibrium concentrations of dye in mg L^−1^. *Q*_e_ is the amount of dye in mg g^−1^ adsorbed onto unit mass of adsorbent at equilibrium. *V* is the volume of dye solution in millilitre (mL); and *m* is the mass of the adsorbent in gram (g).

## 3 Results and discussion

### 3.1 Synthesis and characterization

**Fig. 1:**
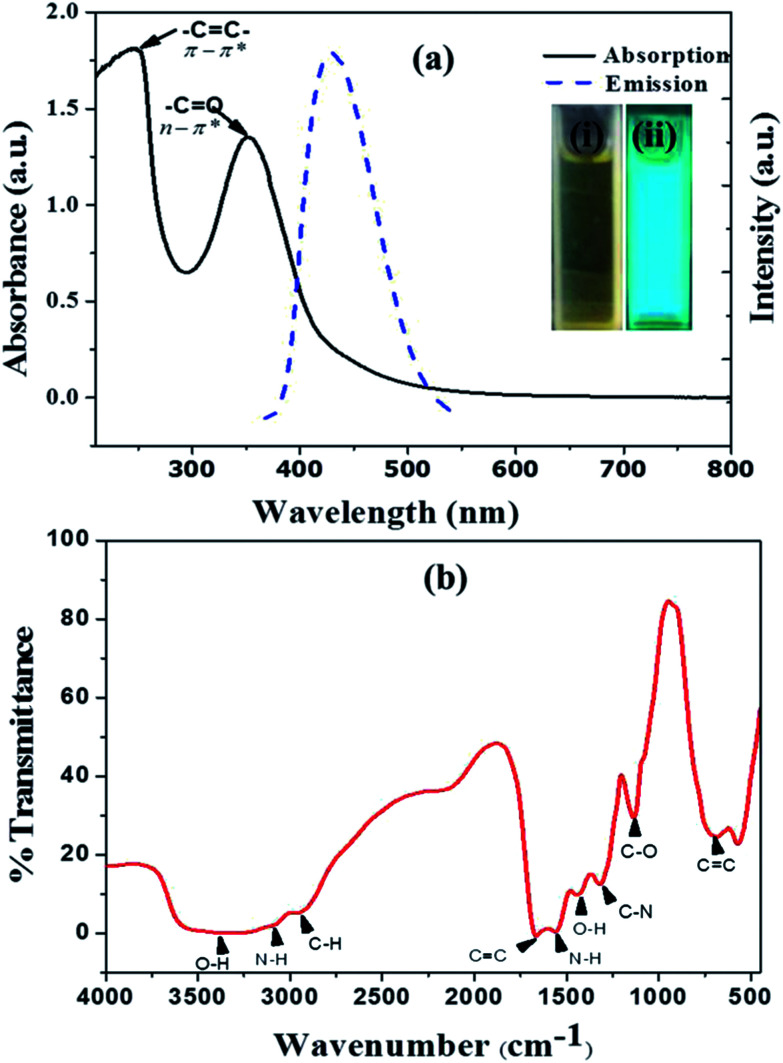
(a) UV-Vis absorption (solid black line) and emission spectra (blue broken line) inset at right (i) NCQDs solution at day light and (ii) NCQDs solution under ultraviolet radiation, (b) FTIR spectrum of NCQDs.

Fluorescent and highly water soluble nitrogen doped carbon quantum dots (NCQDs) were synthesized by hydrothermal method using lemon juice as precursor and ethylenediamine as coreagent (ESI: Fig. ESI1†). In the process, the chemicals present in lemon juice such as citric acid and ascorbic acid undergoes carbonization forming amorphous graphitic carbon dots and then doped and functionalized by ethylenediamine to form NCQDs. The prepared NCQDs exhibits two typical absorption peaks at 245 nm and 353 nm as shown in Fig. 1a (black solid line) which extended with tail to visible region. The first absorption peak at 245 nm could be assigned to π – π* transition of aromatic –C=C– bonds in the sp^2^ hybridized domain of graphitic core and the other peak at 353 nm could be assigned to n – π* transition of –C=O, C–N, or –C–OH bonds which may be from hydroxyl (–COOH) or amine (–NH_2_) groups on surface of NCQDs.^29^ The brown yellowish aqueous solution of NCQDs appears brilliant blue under ultraviolet irradiation (inset in Fig. 1(ii)) which indicate the bright luminescence of the prepared NCQDs. In Fig. 1a (blue broken line) indicate the emission spectra of the blue luminescent NCQDs; excitation at 360 nm and emission at 452 nm.

Information regarding the surface functional group of NCQDs was investigated by Fourier transform infrared spectroscopy (FTIR). As can be seen in the spectrum (Fig. 1b), there are characteristic bands which can indicate the presence of C–O bond, OH, aliphatic C–H, N–H and C–N functional groups. The high water solubility of the NCQDs is as a result of these different functional groups on the surface.^30–32^

**Fig. 2:**
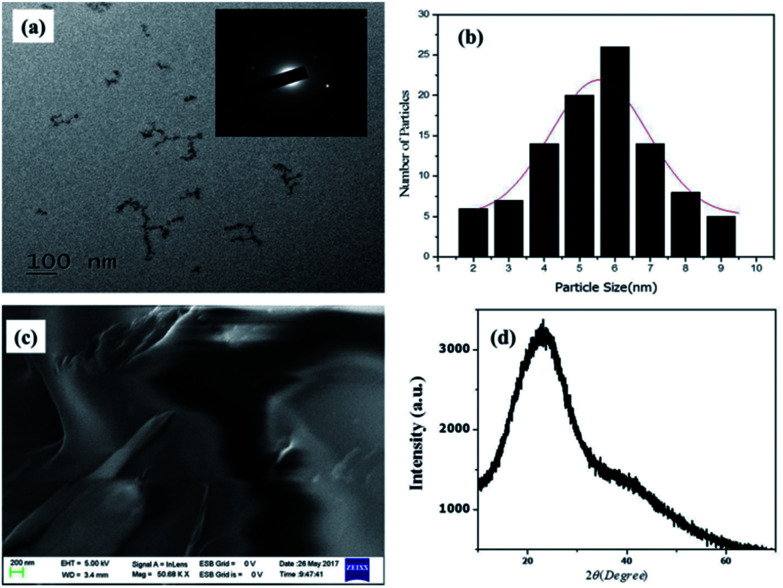
(a) TEM image (inset SAED) (b) histogram of particle size distribution), (c) FESEM image, (d) XRD of NCQDs.

The morphological properties of NCQDs were confirmed by TEM (Fig. 2a)and as the result indicated, the NCQDs particles are well uniformly distributed quasi-spherical nanoparticles with narrow size distribution in diameter range of 2–9 nm with an average of 5.5 nm based on statistical analysis of more than 90 dots (Fig. 2b). The holes in the selected area electron diffraction (SAED) of the NCQDs (inset in Fig. 2 (a)) indicated the particle formation and only two bright spots observed showing the amorphous nature. Paper sheet layer like FESEM image in Fig. 2c confirmed the amorphous nature of NCQDs. Result from elemental composition analysis of EDS spectrum (Fig. ESI3a†) reveal the presence of C, O and N in the as synthesized material indicating well formation of nitrogen doped carbon quantum dots. X-ray diffraction (XRD) patterns show broad intense diffraction peak centered at 2θ = 23° and weak peak at 2θ = 42° which assigned to (002) and (101) diffraction pattern of graphitic carbon, as shown in Fig. 2d which indicates the amorphous nature of the NCQDs.^33,34^

The functional group on NCQDs can play key role in preparing Fe_3_O_4_@NCQDs NCs *via* coprecipitation method, the presence of NCQDs avoid nucleation stage of coprecipitation and hence aggregation free nanocomposite formed.

A possible plausible formation mechanism of Fe_3_O_4_@NCQDs NCs *via* this method is that the carboxylic and amino group on NCQDs chelated with Fe^3+^ and Fe^2+^ to form ferric and ferrous complex. In the presence of NaOH, there is also formation of bonds between OH^−^ and (Fe^2+^, Fe^3+^). With heating, HO^−^⋯Fe^3+^ and OH^−^⋯Fe^2+^ bonds dominate over COO^−^⋯Fe^3+^ and COO^−^⋯Fe^2+^ bonds, and as a result ferric hydroxide, Fe(OH)_3_ and ferrous hydroxide, Fe(OH)_2_ formed. Ferric hydroxide and ferrous hydroxide dehydrated forming magnetite (Fe_3_O_4_) nanoparticle crystals upon heating. The carboxyl and amino group of NCQDs are attached on Fe_3_O_4_ NPs surface through chelation to iron ions. To effectively form Fe_3_O_4_@NCQD NCs, amino and carbonyl functional groups on surface of NCQDs interact with COO^−^⋯Fe^3+^ and COO^−^⋯Fe^2+^ through electrostatic interaction by forming bidentate coordinate covalent bond. As a result stable, relatively aggregate free and uniform sizes Fe_3_O_4_@NCQDs NCs were formed.

**Fig. 3:**
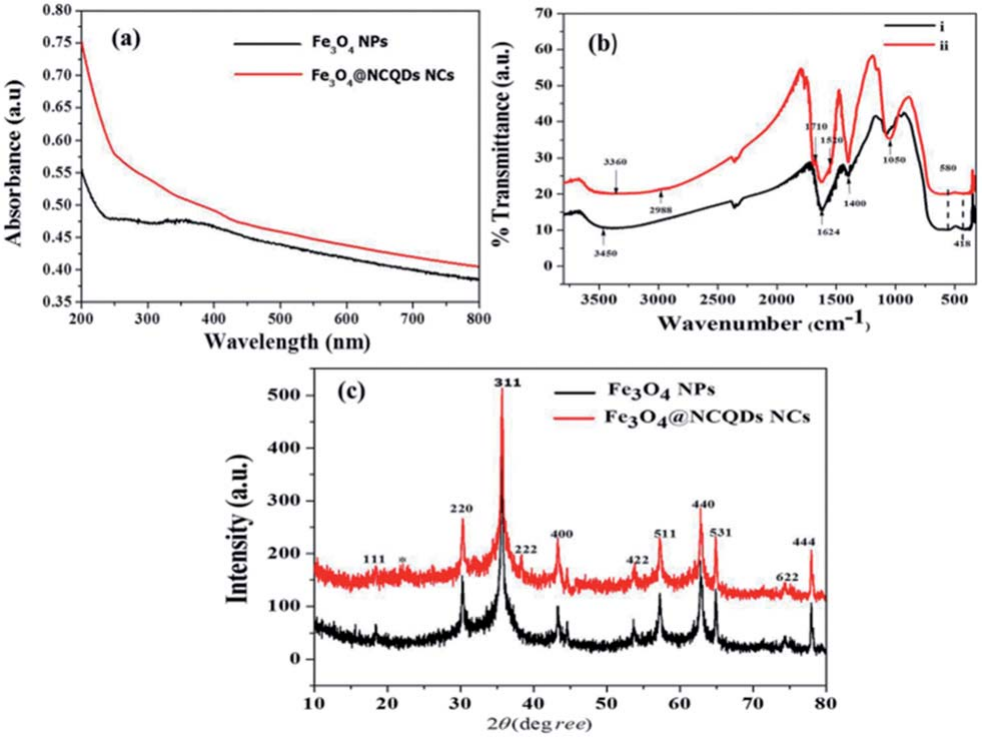
(a) UV-Vis absorption spectra of Fe_3_O_4_@NCQDs NCs and Fe_3_O_4_ NPs, (b) FTIR spectra of Fe_3_O_4_@NCQDs NCs prepared at different proportion of NCQDs solution (i) 10 times diluted & (ii) 5 times diluted, and (c) the powder XRD patterns of Fe_3_O_4_ NPs and Fe_3_O_4_@NCQDs NCs.

Fig. 3a shows the UV-visible absorption spectra of Fe_3_O_4_@NCQDs NCs and Fe_3_O_4_ NPs. The UV-visible spectrum show a broad absorption peak at 350 nm which extended to near IR region, which is primarily due to absorption and scattering of light by Fe_3_O_4_ NPs.^14^ The strong absorption peak around 200 nm for Fe_3_O_4_@NCQDs NCs ascribed to π – π* transition of NCQDs, which indicates effective combining of Fe_3_O_4_ NPs and NCQDs.

The FTIR spectra of Fe_3_O_4_@NCQDs NCs synthesised using different proportion of NCQDs depicted in Fig. 3b. FTIR spectra show broad overlapping band around 3360 cm^−1^, which can be attributed to the *ν* (O–H) and *ν* (N–H) stretching vibration of the hydroxyl and amine group of NCQDs. The band at 1050 cm^−1^ ascribed to the presence of an alcoholic C – O stretching.^35^ The bands at 1624 cm^−1^ and 1400 cm^−1^ are ascribed to asymmetric (*ν*_as_) and symmetric (*ν*_s_) stretching of the COO^−^ respectively.^36^ The band at 1624 cm^−1^ is also due to the N – H bending mode of the amine group coupling with the *ν*_as_ C – O. The energy difference (Δ*ν*) between the *ν*_as_ (COO^−^) and *v*_s_ (COO^−^) IR bands can reveal the interaction between the carboxylate head and the metal atom.^37^ The Δ*v* (1624−1400 = 224 cm^−1^) is ascribed to bridging and bidentate coordination, where the interaction between the COO^−^ group and the Fe atom was covalent.^38^ The characteristic absorption peaks for Fe_3_O_4_ NPs were observed at 580 cm^−1^ and 418 cm^−1^ which ascribed to the stretching vibrations of Fe^2+^ – O and Fe^3+^ – O bonds for Fe_3_O_4_ NPs respectively.^39^

The crystallite structure of the as prepared NCQDs, Fe_3_O_4_ NPs and Fe_3_O_4_@NCQDs NCs were determined by using X-ray diffraction (XRD) technique. Fig. 3d shows the XRD pattern of Fe_3_O_4_ NPs and Fe_3_O_4_@NCQDs NCs. The XRD peaks at 2*θ* of 18.48°, 30.32°, 35.64°, 38.36°, 43.38°, 53.76°, 57.32°, 62.78°, 64.88°, 74.30° and 77.98° correspond to the diffraction crystallite planes of (111), (220), (311), (222), (400), (422), (511), (440), (530), (622) and (444) respectively in which all peaks indexed to the inverse spinal phase of magnetite (JCPDS file, no. 19-0629). In addition to Fe_3_O_4_ NPs patterns, in the XRD pattern of Fe_3_O_4_@NCQDs NCs (Fig. 3c), there is an additional weak peak at 22.64° which is characteristic of graphitic NCQDs and can be indexed to the (002) reflection indicating good binding of Fe_3_O_4_ NPs and NCQDs in formation of Fe_3_O_4_@NCQDs NCs. The sharp and strong peaks indicate high crystallinity of the as synthesized Fe_3_O_4_@NCQDs NCs.^40^

**Fig. 4:**
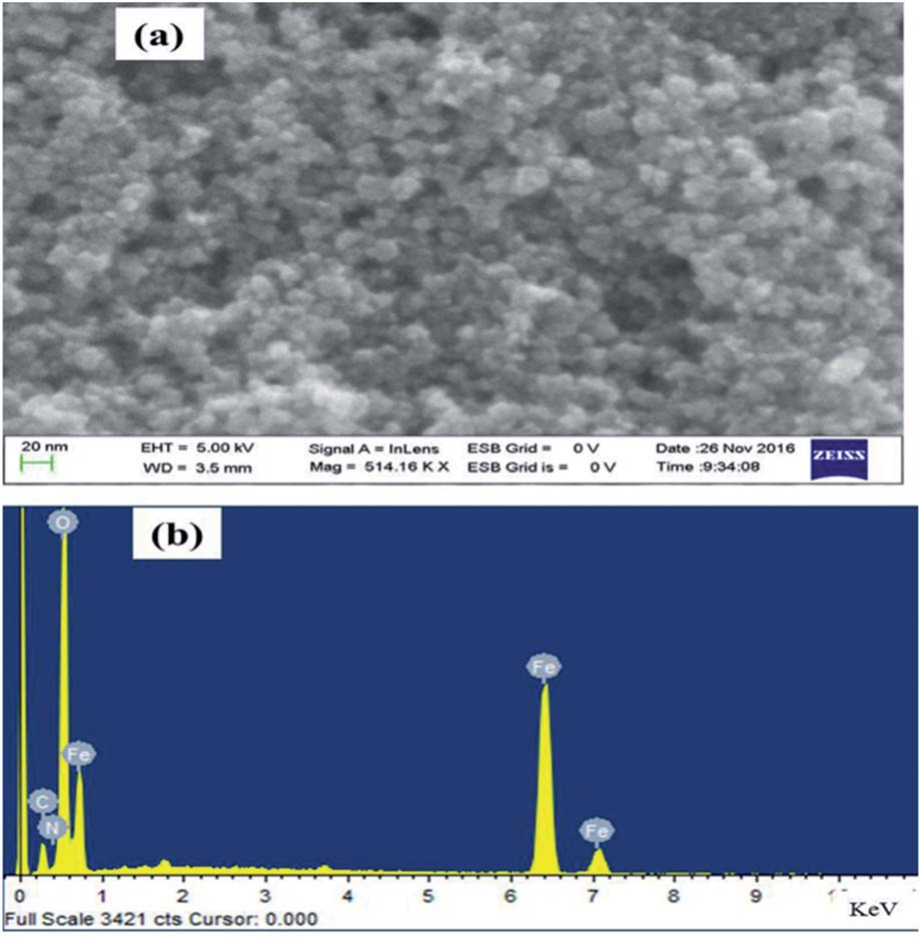
(a) FESEM image and (b) EDX spectra of Fe_3_O_4_@NCQDs NCs.

Morphological study of the synthesised Fe_3_O_4_@NCQDs NCs was investigated using FESEM and TEM. As depicted in Fig. 4, FESEM images clearly showed that the Fe_3_O_4_@NCQDs NCs have nearly spherical shape with uniform distribution. The presence of iron (Fe), oxygen (O), carbon(C) and nitrogen (N) in EDS spectrum confirms the successful formation of Fe_3_O_4_@NCQDs NCs.

**Fig. 5:**
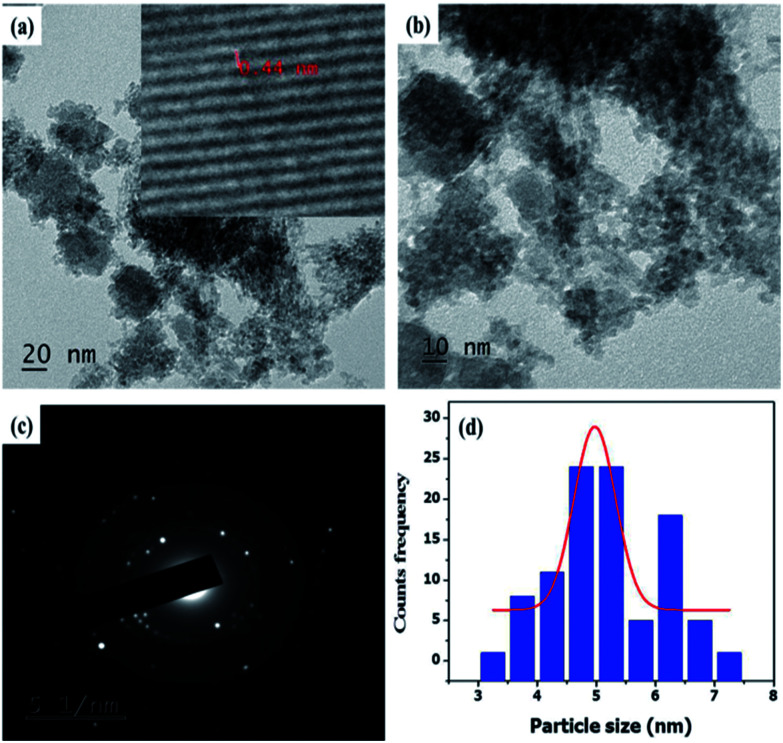
(a and b) Representative TEM images (c) SAED and (d) particle size distribution of Fe_3_O_4_@NCQDs NCs.

The representative TEM images of Fe_3_O_4_@NCQDs NCs were presented in Fig. 5a and b. It is clear from TEM images that Fe_3_O_4_@NCQDs NCs have spherical shape without any aggregation. The particle size distribution of the as synthesized nanocomposites is shown in histogram (Fig. 5d) and the calculated average particle size based on over 100 particles is 5 nm. In addition, crystalline diffraction rings from the selected area diffraction (SEAD) patterns (Fig. 5c) demonstrated that the crystalline nature of the as prepared Fe_3_O_4_@NCQDs NCs. The inset in Fig. 5a obtained from HRTEM indicates the lattice space (0.44 nm) which is comparable to XRD results.

### 3.2 Optimization of adsorption parameters

#### 3.2.1 Effect of initial concentration

**Fig. 6:**
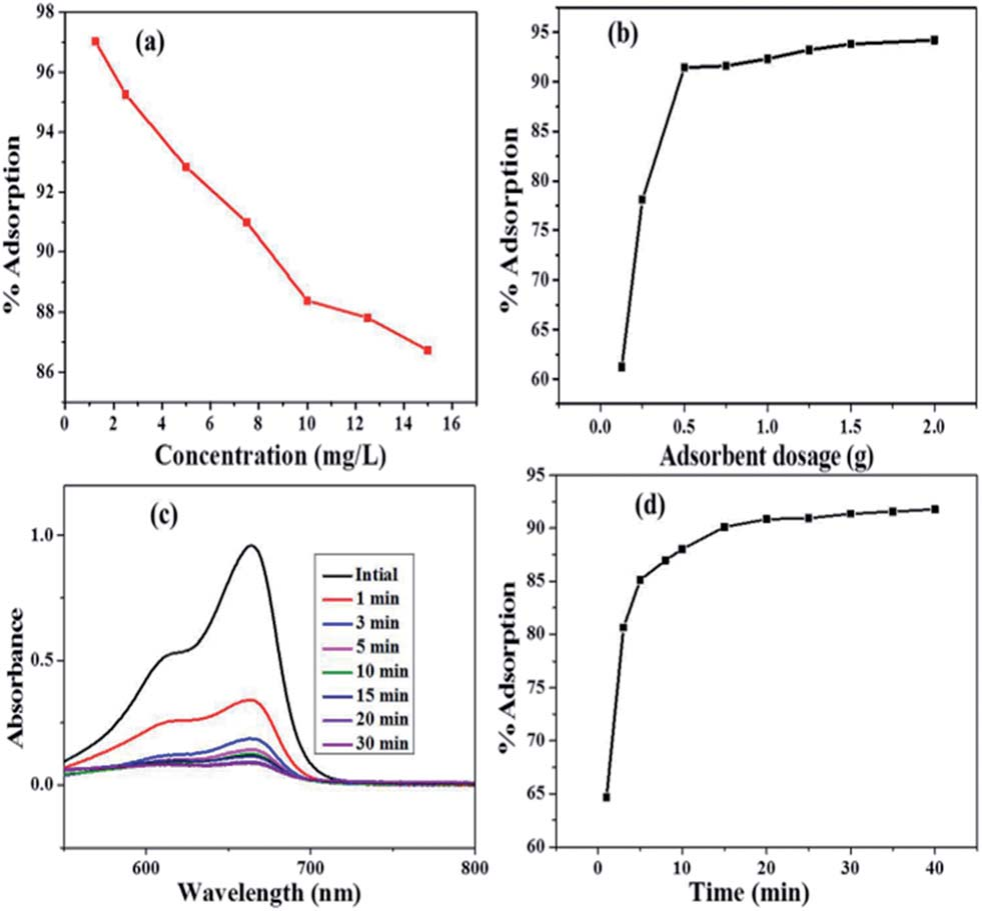
(a) Effect of initial MB dye concentration on adsorption (b) effect of adsorbent dosage on adsorption (c) UV-Vis absorption spectrum and (d) % adsorbance of MB dye (7.5 mg L^−1^) on Fe_3_O_4_@NCQD NCs (adsorbent dose = 50 mg, temperature = 298 K, pH = 11) at different time interval.

To optimize this parameter, different concentrations (1.25–15 mg L^−1^) of 100 mL MB dye solution were used at (contact time = 20 min), (pH = 11), (adsorbent dose = 50 mg) and (temperature = 298 K). The effect of varying concentrations on adsorption is shown in Fig. 6a. The dye removal efficiency of Fe_3_O_4_@NCQDs NCs was dependent on the initial concentrations of the dye solution in that the maximum adsorption took place at 1.25 mg L^−1^, which decreased up to 15 mg L^−1^ from 97.01 to 86.72% with increasing adsorbate concentration. The decrease in adsorption with an increase in dye concentration could be explained on the basis that MB removal depends on the availability of the binding sites on the Fe_3_O_4_@NCQDs NCs adsorbent surface. Total available adsorption sites for a fixed amount of Fe_3_O_4_@NCQDs NCs were used at 7.5 mg L^−1^ concentration and therefore, 7.5 mg L^−1^ was taken as optimum initial concentration.

#### 3.2.2 Effect of adsorbent dosage

Optimization of dosage was carried out using 0.1–2.0 g L^−1^ of Fe_3_O_4_@NCQDs NCs. MB dye concentration, pH, contact time and temperature were 7.5 mg L^−1^, 11.0, 20 min and 298 K, respectively. The effect of dose on percept uptake of MB onto Fe_3_O_4_@NCQDs NCs is shown in Fig. 6b, indicating rapid increase in adsorption with increasing doses. MB adsorption increased from 60.28 to 94.49% at dosages of 0.1 and 2.0 g L^−1^ of Fe_3_O_4_@NCQDs NCs adsorbent. Adsorption was 90.84% at 0.5 g L^−1^ and on further increasing the dose, adsorption percentage was slightly increased with no significance. Therefore, 0.5 g L^−1^ of Fe_3_O_4_@NCQDs NCs was considered as optimum dosage.

#### 3.2.3 Effect of contact time

Time optimization for the maximum removal of MB dye onto Fe_3_O_4_@NCQDs NCs adsorbent was done by varying contact time (1–40 min). The initial concentration, adsorbent dosage, pH and temperature were 7.5 mg L^−1^, 0.5 g L^−1^, 11.00 and 298 K, respectively. The result showed that adsorption of the MB dye onto Fe_3_O_4_@NCQDs NCs adsorbent consisted of two phases; initial stage consisting of rapid adsorption (0–15 min) and final stage with the relatively slow adsorption rate (Fig. 6c and d). The adsorption increased from 64.6 to 90.84% as the contact time was increased from 1 to 20 min. On further increasing the contact time up to 40 min adsorption increased to 91.78%, but this increase was insignificant and slow. Therefore, 20 min was considered to be the optimum time for MB dye adsorption onto Fe_3_O_4_@NCQDs NCs.

#### 3.2.4 Effect of pH

**Fig. 7:**
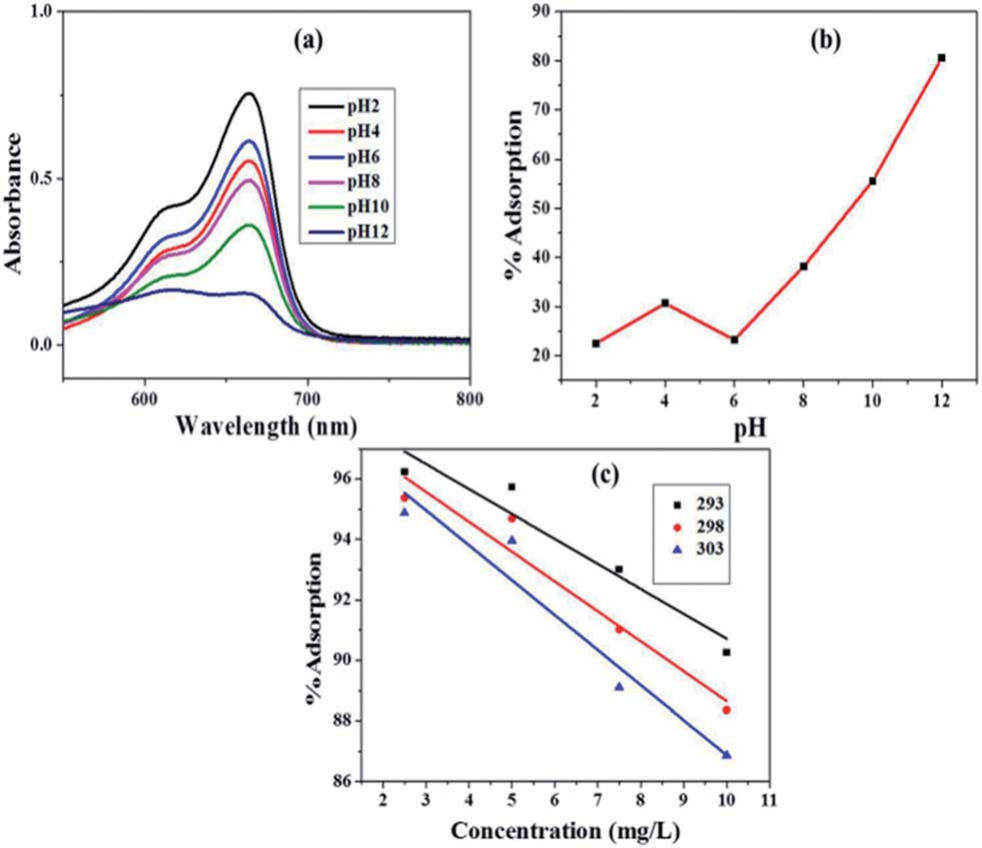
(a) UV-Vis absorption spectra at different pH (b) effect of pH on adsorption and (c) effect of temperature at different concentration on adsorption of MB on Fe_3_O_4_@NCQDs NCs.

One of the most governing factors for removal of dye from water using adsorption process is pH.^41^ The effect of varying pH (2.0 to 12.0) on the adsorption of MB onto Fe_3_O_4_@NCQDs NCs was investigated with initial dye concentration of 7.5 mg L^−1^, catalyst dosage of 0.5 g L^−1^, and contact time of 20 min at 298 K (Fig. 7a and b). The pH of solution was adjusted using 0.1 M HCl/NaOH. The adsorption capacity increased continuously as the pH increased from 2–12. At acidic pH, lower adsorption of MB was observed due to the presence of excess H_3_O^+^ ions competing with MB cations for the available adsorption sites which reduce the adsorbed amount. Therefore, pH of 11 was selected as optimum pH for adsorption of MB dye solution onto Fe_3_O_4_@NCQDs NCs.

#### 3.2.5 Effect of temperature

The adsorption studies were carried out at 293, 298 and 303 K, and the results of these experiments are presented in Fig. 7c. The solution temperature was controlled using water bath by using ice as cooling agent. The adsorption decreased almost for all concentrations of methyl blue when temperature was raised from 293 to 303 K. The decrease in adsorption with rise of temperature indicated exothermic nature of the adsorption process.

### 3.3 Isotherm modelling of adsorption

The adsorption data were analyzed by fitting to isotherm models that are Langmuir, Freundlich and Temkin. The isotherm experiments were carried out at 293, 298 and 303 K with 100 mL MB solution of 2.5–10 mg L^−1^ concentrations, at solution pH 11.0 and adsorbent dosage of 0.5 g L^−1^.

#### 3.3.1 Langmuir isotherm model

**Fig. 8:**
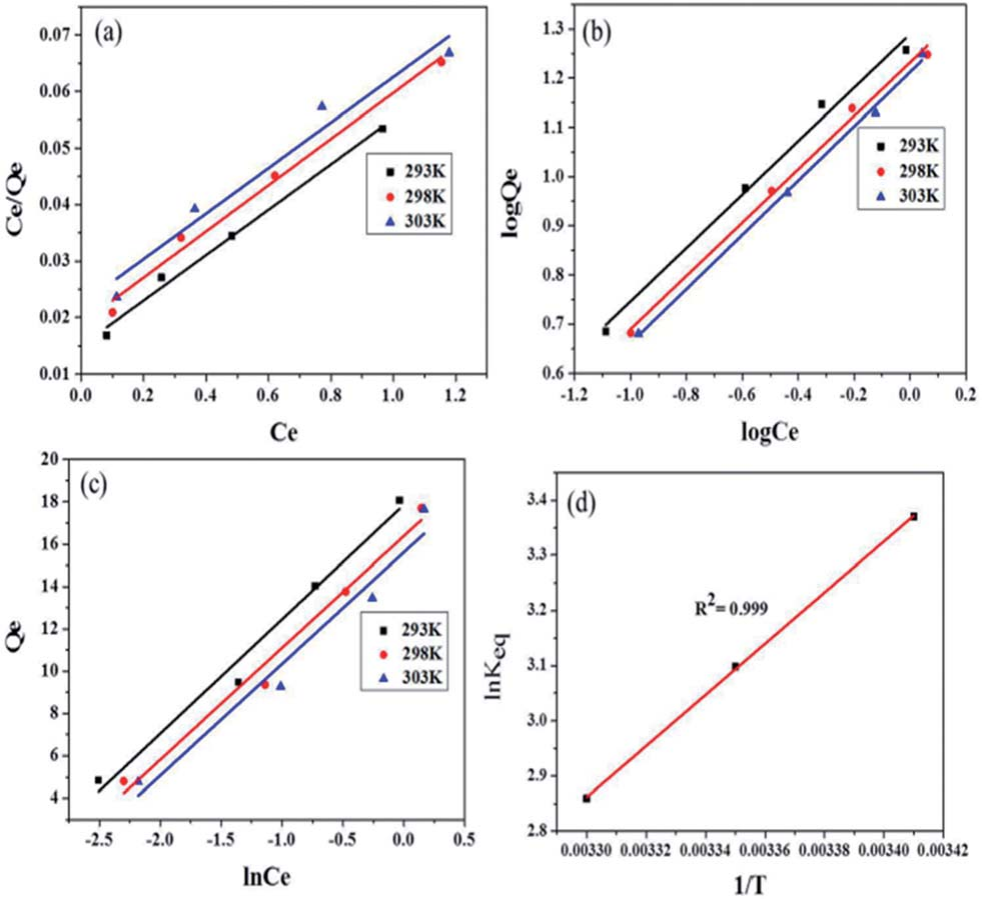
(a) Langmuir adsorption isotherm plot (b) Freundlich adsorption isotherm plot (c) Temkin adsorption isotherm plot (d) thermodynamic plot of MB dye adsorption onto Fe_3_O_4_@NCQDs NCs.

The Langmuir model assumes that uptake of adsorbate occurs on a homogeneous surface by monolayer adsorption and that there is no interaction between adsorbent and adsorbate species.^42,43^ Langmuir mathematical equation is:

3 *C*_e_/*Q*_e_ = *C*_e_/*Q*_0_ + 1/*Q*_0_*b*

where, *C*_e_ (mg L^−1^) and *Q*_e_ (mg g^−1^) have the usual meanings. *Q*_0_ (adsorption capacity in mg g^−1^) is the amount of adsorbate that can be absorbed by a unit mass of the adsorbent for the formation of monolayer on the surface and ‘*b*’ is Langmuir constant, which is related to the affinity between the adsorbent and adsorbate. Both ‘*b*’ and ‘*Q*_0_’ are characteristics of adsorbent and adsorbate pair. The plot of *C*_e_/*Q*_e_*vs. C*_e_ at 293, 298 and 303 K is shown in Fig. 8a from which ‘*Q*_0_’and ‘*b*’ values were evaluated from the slope and intercept of the plots. The values of ‘*Q*_0_’ were observed to be 24.888, 24.480 and 24.414 mg g^−1^ at 293, 298 and 303 K, respectively. These values slightly decreased with temperature, which indicated exothermic adsorption of MB onto Fe_3_O_4_@NCQDs NCs. The decrease in Langmuir constant, ‘*b*’ values from 2.679 to 1.834 with increase in temperature from 293 to 303 K indicated lower affinity of MB for Fe_3_O_4_@NCQDs NCs at higher temperature. The close to unity values of the regression coefficient, *R*^2^ (0.946–0.989) indicated good fittings of Langmuir isothermal model (ESI: Table ESI1†).

A separation factor which also known as dimensionless equilibrium parameter, *R*_L_ and its value indicates the adsorption nature: unfavorable if *R*_L_ > 1, linear if *R*_L_ = 1, favourable if 0 < *R*_L_ < 1.^44^*R*_L_ was calculated using the following equation:

4 *R*_L_ = 1/(1 + *bC*_e_)

The calculated value of *R*_L_ at 293, 298 and 303 K are 0.436, 0.428 and 0.414, respectively, indicating the suitability of Fe_3_O_4_@NCQDs NCs for adsorption of MB dye solution from waste water.

#### 3.3.2 Freundlich isotherm model

The Freundlich model assumes that the uptake of adsorbate occurs on a heterogeneous adsorbent surface.^45^ The mathematical equation of Freundlich isotherm is expressed as:

5 *Q*_e_ = *K*_F_*C*_e_^1/*n*^

6 log *Q*_e_ = log *K*_F_ + 1/*n*(log *C*_e_)

Freundlich constants, *K*_F_ (adsorption capacity), and *n* (adsorption intensity) calculated from the slopes and intercepts of the Freundlich plots, log *Q*_e_*vs.* log *C*_e_ (Fig. 8b). Magnitude of ‘*K*_F_’ can be taken as a relative measure of adsorption capacity of Fe_3_O_4_@NCQDs NCs for the adsorption of MB. The Freundlich constant *n* (intensity of adsorption) varies with the heterogeneity of the adsorbent and for favourable adsorption ‘*n*’ values should be in the range 1–10.^46,47^

The values of Freundlich constant ‘*n*’ at 293, 298 and 303 K were 1.838, 1.842, and 1.811 are higher than unity suggesting feasibility of adsorption of MB onto the surface of Fe_3_O_4_@NCQDs NCs. The values of ‘*K*_F_’ were 19.491, 17.069 and 16.317 mg g^−1^, which clearly showed that ‘*K*_F_’ decreased slightly from 293 to 303 K, indicating the decrease in the adsorption capacity at higher temperature. This is in agreement with Langmuir isotherm observations. The regression coefficients were more close to unity as compared to that of Langmuir isotherm showing better fitting of the Freundlich model, which suggest adsorption of MB onto heterogeneous Fe_3_O_4_@NCQDs NCs surface.

#### 3.3.3 Temkin isotherm model

Temkin isotherm is based on the assumption that the heat of adsorption of all molecules in layer decreases linearly with coverage of adsorbent surface due to adsorbate–adsorbent interactions.^48^ The mathematical equation of the isotherm is expressed as:

7 *Q*_e_ = *RT*/*b*_T_(ln *A*_T_ + ln *C*_e_)

where, *A*_T_ (L g^−1^) and *b*_T_ (kJ mol^−1^) are Temkin isotherm constants, and ‘*R*’ and ‘*T*’ are the universal gas constant and absolute temperature (K), respectively. ‘*A*_T_’ is the equilibrium binding constant, related to the maximum binding energy and ‘*b*_T_’ is a constant related to the heat of adsorption.^49^

**Fig. 9:**
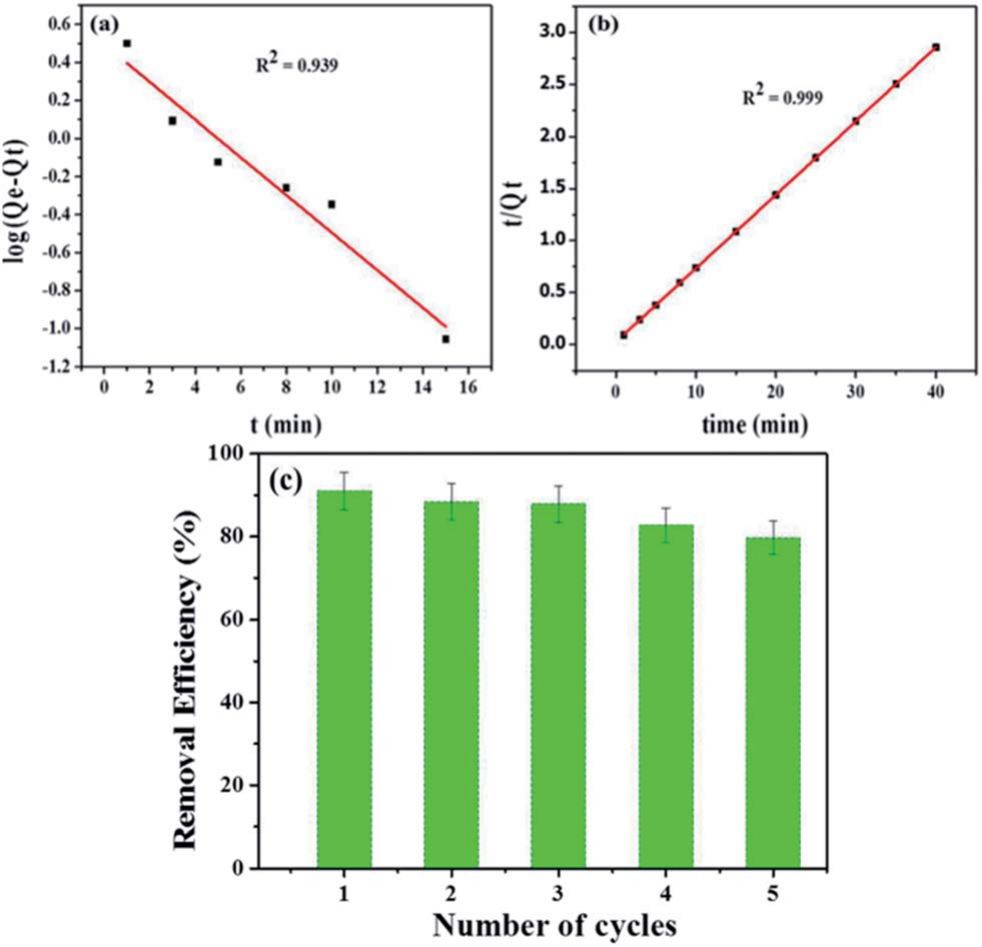
(a) Pseudo-first order kinetic plot (b) pseudo-second order kinetic plot and (c) The recyclability of the Fe_3_O_4_@NCQDs NCs in the MB dye removal from aqueous solution for 5 successive cycles (Fe_3_O_4_@NCQDs NCs = 50 mg and MB = 7.5 ppm).

The plot of *Q*_e_*vs.* ln *C*_e_ is shown in Fig. 9c. The constants ‘*b*_T_’ and ‘*A*_T_’ were calculated from the slope and intercept of the plot, respectively, and are listed in ESI: Table ESI1† along with regression coefficients. High magnitudes of ‘*A*_T_’ and ‘*b*_T_” indicated high interactions between MB and adsorbent. Therefore, the process might be chemisorption. The values of *R*^2^ were well close to the unity showing good fitting of the adsorption data to Temkin isotherm model.

### 3.4 Thermodynamic studies of adsorption

Thermodynamic studies are used to describe any reaction in a better way. In this work, thermodynamic studies were performed and the thermodynamic parameters (Δ*G*°, Δ*H*°, and Δ*S*°) were determined at 293, 298 and 303 K temperature using the equations:

8 *K*_eq_ = *Q*_e_/*C*_e_

9 Δ*G*° = −*RT* ln *K*_eq_

10 ln *K*_eq_ = −Δ*H*°/*RT* + Δ*S*°/*R*

where *Q*_e_ is solid phase concentration at equilibrium (mg g^−1^), *C*_e_ is equilibrium concentration of dye in solution (mg L^−1^) and *K*_eq_ is equilibrium constant. The calculated free energy change (Δ*G*°), enthalpy change (Δ*H*°) and entropy change (Δ*S*°) parameters at different temperatures are presented in ESI: Table ESI2.† Δ*H*° and Δ*S*° values are calculated from the slope and intercept of the linear plots of ln *K*_eq_*vs.* 1/*T*, respectively (Fig. 8d). Negative values of Δ*G*° indicated spontaneity and feasibility while negative values of entropy change Δ*S*° and enthalpy change Δ*H*° indicated the exothermic nature of adsorption of MB dye solution onto Fe_3_O_4_@NCQDs NCs.

### 3.5 Kinetics and mechanism of MB dye adsorption studies onto Fe_3_O_4_@NCQDs NCs

The kinetics of adsorption which shows the rate of transport of the dye from solution to the surface of adsorbent was investigated by pseudo-first-order and pseudo-second order kinetic models.

Linearized mathematical form of pseudo-first order model^50^

11 log(*Q*_e_ − *Q*_*t*_) = log *Q*_e_ − (*k*_1_/2.303) × *t*

where *k*_1_ (min^−1^) is pseudo-first order rate constant, and *Q*_*t*_ is the amount (mg g^−1^) of adsorbate on the adsorbent surface at time *t*. The slope and intercept of straight line plots of log(*Q*_e_ − *Q*_*t*_) *vs. t* (Fig. 9a) gave the values of (*k*_1_ = 0.228 min^−1^) and *Q*_e_(3.125 mg g^−1^), respectively, and are tabulated in ESI: Table ESI3.†*R*^2^ = 0.939 which is close to unity indicating fitness of pseudo-first order model.

Linearized mathematical form of pseudo-second order model^51^

12 *t*/*Q*_*t*_ = 1/*h* + *t*/*Q*_e_

where *h* is initial rate constant (*h* = *k*_2_*Q*_e_^2^) and *k*_2_ is overall pseudo-second order constant.

According to the values of correlation coefficient and *Q*_e_(calc.) (ESI: Table ESI3†) and Fig. 9b, the pseudo-second order model is found to be more suitable to describe the adsorption kinetic data than the pseudo-first order model for adsorption of MB dye onto Fe_3_O_4_@NCQDs NCs. Hence, it has been confirmed that the adsorption process follows pseudo-second order kinetic behaviour.

### 3.6 Recyclability of adsorbent

Fe_3_O_4_@NCQDs NCs was used as adsorbent for removal of MB from aqueous solution. The used Fe_3_O_4_@NCQDs NCs can be easily separated from solution at end of application using external magnet. Recyclability of the Fe_3_O_4_@NCQDs NCs in MB removal was tested for 5 times and the results are depicted in Fig. 9c. The desorption process of MB was conducted by adjusting pH to 2 using 0.1 M HCl to remove MB form surface of Fe_3_O_4_@NCQDs NCs, then washing with acetone. The regenerated Fe_3_O_4_@NCQDs NCs adsorbent was used to evaluate the reusability of the Fe_3_O_4_@NCQDs NCs for adsorption times. The removal efficiency for the first time 90.8% decreases to 79.2% at the fifth cycle. Our results suggested that the Fe_3_O_4_@NCQDs NCs can be reused over 5 times.

## 4 Conclusion

In conclusion, we have developed a cost effective method to prepare spherical shaped Fe_3_O_4_@NCQDs NCs using lemon extract as precursor. Superparamagnetic Fe_3_O_4_@NCQDs NCs have spherical morphology with an average particle size of 5 nm. Fe_3_O_4_@NCQDs NCs were used as adsorbent removal of MB dye pollutant from aqueous solution. Batch adsorption experiments showed enhanced rapid removal of MB dye within 20 minutes with adsorption efficiency of about 90.84% at optimum conditions. The adsorption data showed good fitting to Freundlich with *R*^2^ of 0.993 at 298 K temperature. The Freundlich constant ‘*n*’ value at 298 K was 1.842 which suggested the feasibility of adsorption of MB onto the surface of Fe_3_O_4_@NCQDs NCs. Thermodynamic studies indicated the spontaneity and exothermic nature of the adsorption process. From kinetics study, it was found that, the pseudo-second order model is more suitable to describe the adsorption kinetic data than the pseudo-first order model for adsorption of MB dye onto Fe_3_O_4_@NCQDs NCs.

## Conflicts of interest

There are no conflicts to declare.

## Supplementary Material

RA-008-C8RA00158H-s001
