# Supplementary material for: Synthesis of nitrogen doped carbon quantum dots/magnetite nanocomposites for efficient removal of methyl blue dye pollutant from contaminated water
Source: RSC Adv. 2018 Feb 23;8(16):8528–36. doi: 10.1039/c8ra00158h (PMC9078528; doi:10.1039/c8ra00158h)
Supplement: RA-008-C8RA00158H-s001 [file RA-008-C8RA00158H-s001.pdf]

Electronic Supplementary Material (ESI) for RSC Advances.
This journal is © The Royal Society of Chemistry 2018

Synthesis of Nitrogen Doped Carbon Quantum Dots/Magnetite nanocomposites for efficient removal of methyl blue dye pollutant from contaminated Water

Aschalew Tadesse[a, c], Dharmasoth Rama Devi[b], Mabrahtu Hagos[a,d], GangaRao Battu[b] and K. Basavaiah[a]*

[a]*Department of Inorganic and Analytical Chemistry, Andhra University, Visakhapatnam-530003, India,*

[b]*AU College of Pharmaceutical Sciences, Andhra University, Visakhapatnam-530003, India*

[c]*Department of Applied Chemistry, Adama Science and Technology university, Adama-1888, Ethiopia*

[d]*Faculty of Natural and Computational Sciences, Woldia University, Woldia-400, Ethiopia*

**Corresponding Author Email: klbasu@gmail.com, Phone: +919908036203*

Supplementary Information

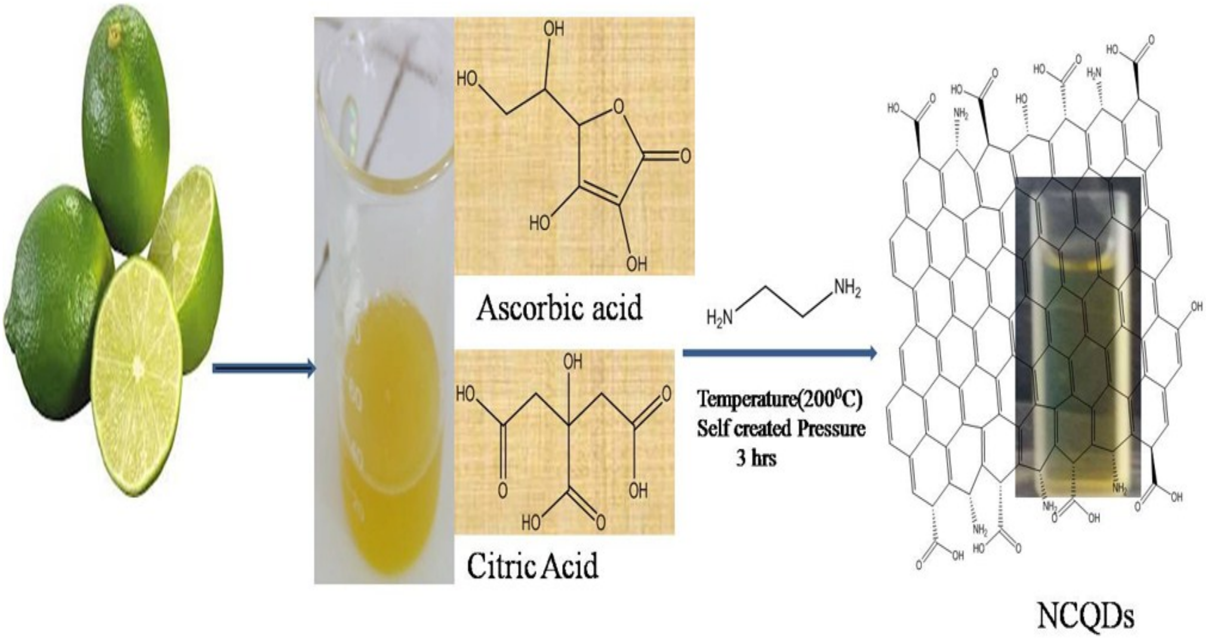

Fig. ESI1: schematic representation of hydrothermal synthesis of NCQDs using lemon juice as precursor and ethylinediamine as coreagent

As seen from Fig.ESI2 (a and b), the sample shows strong fluorescence with symmetrical peaks. Emission intensity increases as excitation wavelength increase from 310 nm to 360 nm then start decreasing and maximum emission intensity obtained at excitation wavelength of 360 nm. Fig.ESI2(d) indicate excitation and emission contour map of NCQDs which showed

multicolour emission of NCQDs. The photoexcitation of nitrogen doped carbon quantum dots may be due to π-plasmon absorption in the core carbon nanoparticles[1] and to large extent pholominescence significantly affected by the surface chemistry of the NCQDs.[2] The as synthesized NCQDs show high FL quantum yield (QY ~31%) relative to the standard Quinine sulphate.

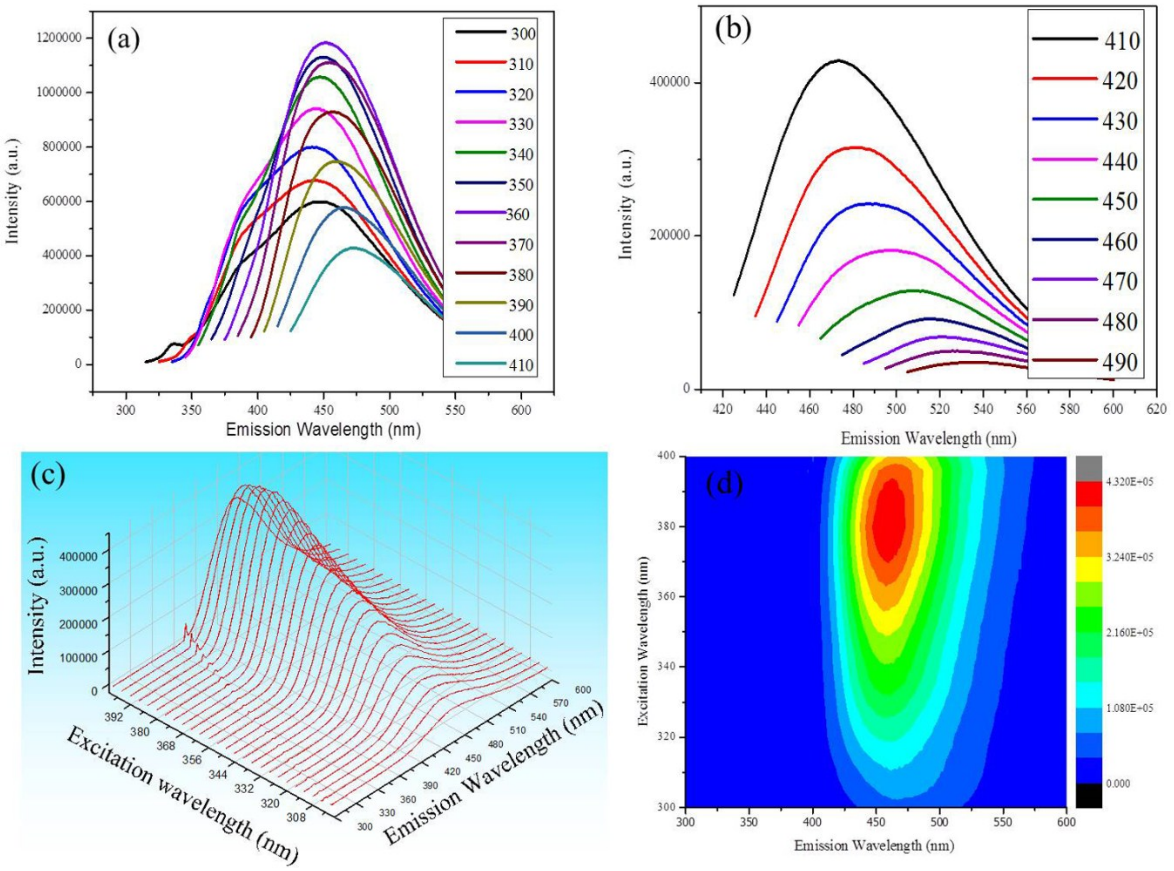

Fig. ESI2 (a) Fluorescence emission spectra of NCQDs under excitation wavelength (300 to 410) (b) excitation wavelength (410 to 490) (c) 3D Fluorescence spectra of NCQDS (d) Excitation and emission contour map of NCQDs

Result from elemental composition analysis of EDX spectrum (Fig.ESI3a) reveal the presence of C, O and N in the as synthesized material indicating well formation of nitrogen doped carbon quantum dots.

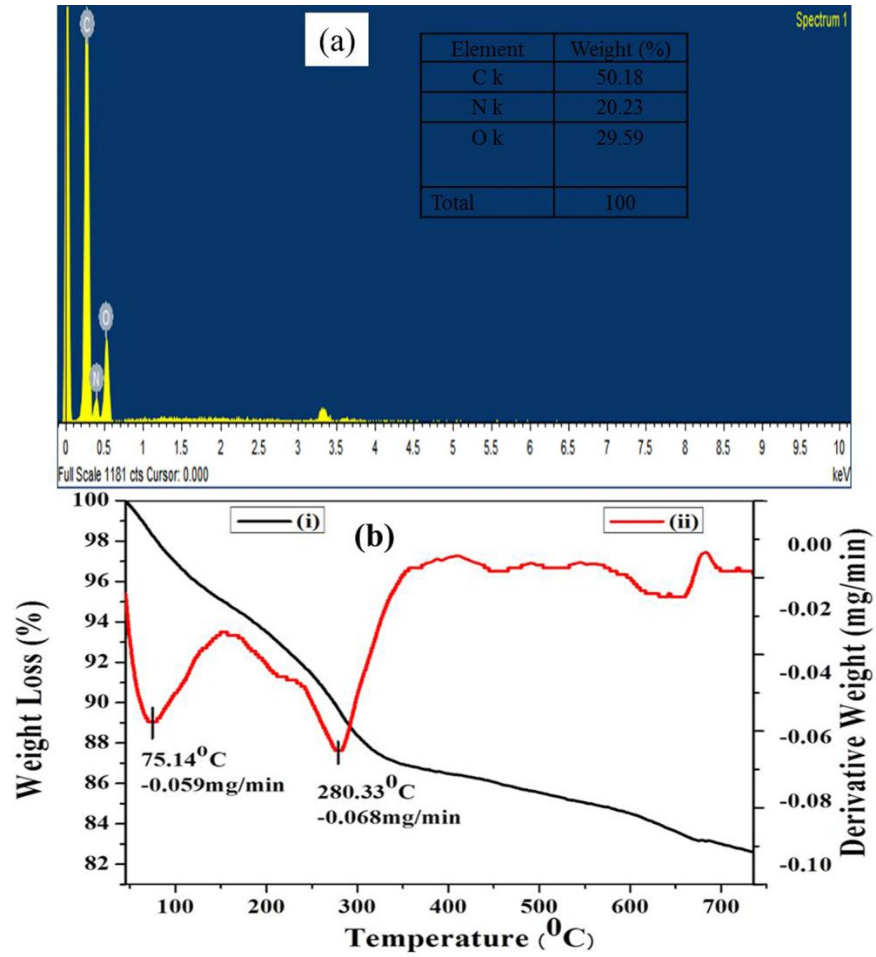

Fig. ESI3 (a) EDS spectraof NCQDs (b) ((i) Thermogravimetric analysis (TGA) and (ii) differential thermal analysis (DTA) of Fe_3O_4@NCQDs NCs

Scheme ESI1 A plausible formation mechanism of Fe3O4@NCQD NCs

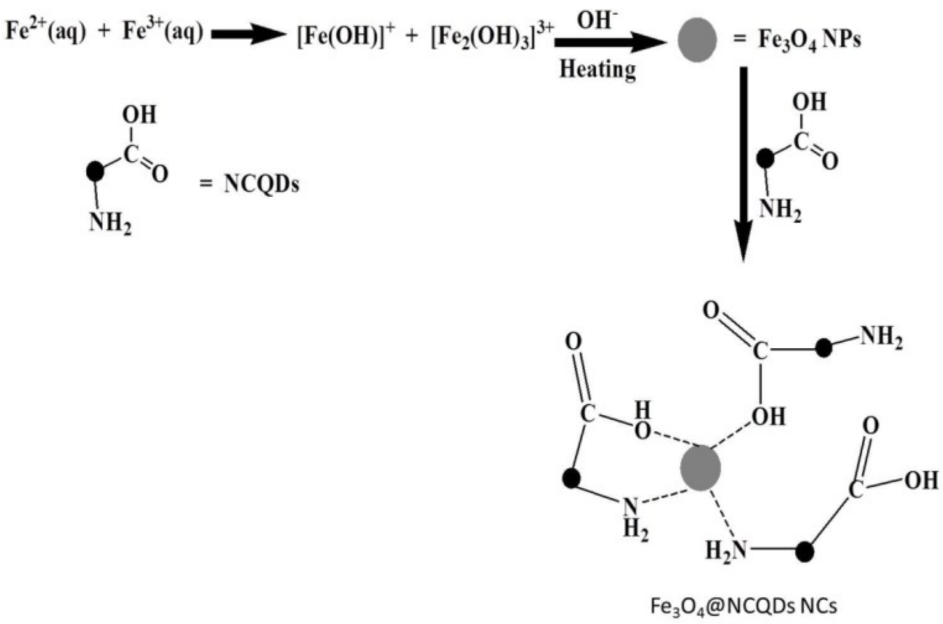

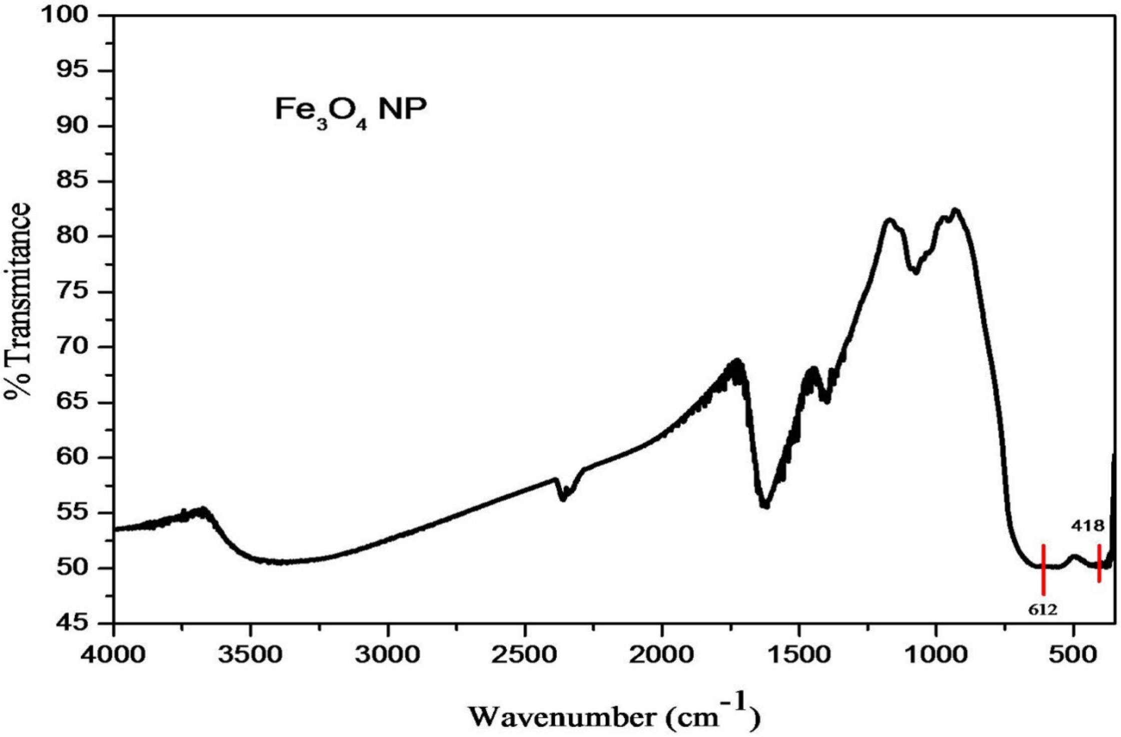

Fig. ESI4 FTIR spectrum of Fe_3O_4 NPs

As shown in Fig.3c, the TGA-DTA of Fe_3O_4@NCQDs NCs has three characteristic decomposition stages. Weight loss (~6.0 %) of Fe_3O_4@NCQDs NCs in the first stage was due to the loss of water, and weight loss (~13.6 %) in the second stage was due to the decomposition of the amine, hydroxyl and carbonyl groups on the surface of NCQDs in nanocomposites structure. In Fig.ESI3b(i), (DTA curve) three peaks were observed: peak between 39 and 150 °C with a maximum at 75.14 °C, peak between 150 and 405 °C with a maximum at 280.33 °C, and the third peak between 404 and 735 °C with a maximum at 650 °C which correspond to three stage transition of the Fe_3O_4@NCQDs NCs. The first stage transition could be due to the loss of water, while the second was due to decomposition of the amine, hydroxyl and carbonyl groups on the surface of carbon quantum dots and third attributed to the arrangement of the structure of the Fe_3O_4@NCQDs NCs.[3]

Magnetic property of material is one of the important properties which decide the easy separation of the materials after environmental remediation. The magnetic properties of the synthesised Fe_3O_4@NCQDs NCs have been investigated by studying the field dependence magnetization at room temperature using vibrating sample magnetometer (VSM) in the range of -15 kOe to 15 kOe applied magnetic field. Fig.ESI5 shows the M–H curves of Fe_3O_4@NCQDs NCs measured at 300 K and the forward and backward magnetization curves overlap completely.

Fig.ESI5 inset in up left side showed almost zero hysteresis loop in narrow range from -0.5 kOe to 0.5 kOe indicating their superparamagnetic behaviour.[4] The saturation magnetization (Ms) value of Fe_3O_4@NCQDs NCs is 24.6 emug−1. The reduced Ms of the Fe_3O_4@NCQDs NCs in comparison to Ms of bulk magnetite is possibly due to the presence of non-magnetic NCQDs with the magnetic ordering of the iron oxide nanosystem.[5] In Fig.ESI5 inset right bottom showed the easy separation of Fe_3O_4@NCQDs NCs from solution by using external magnet.

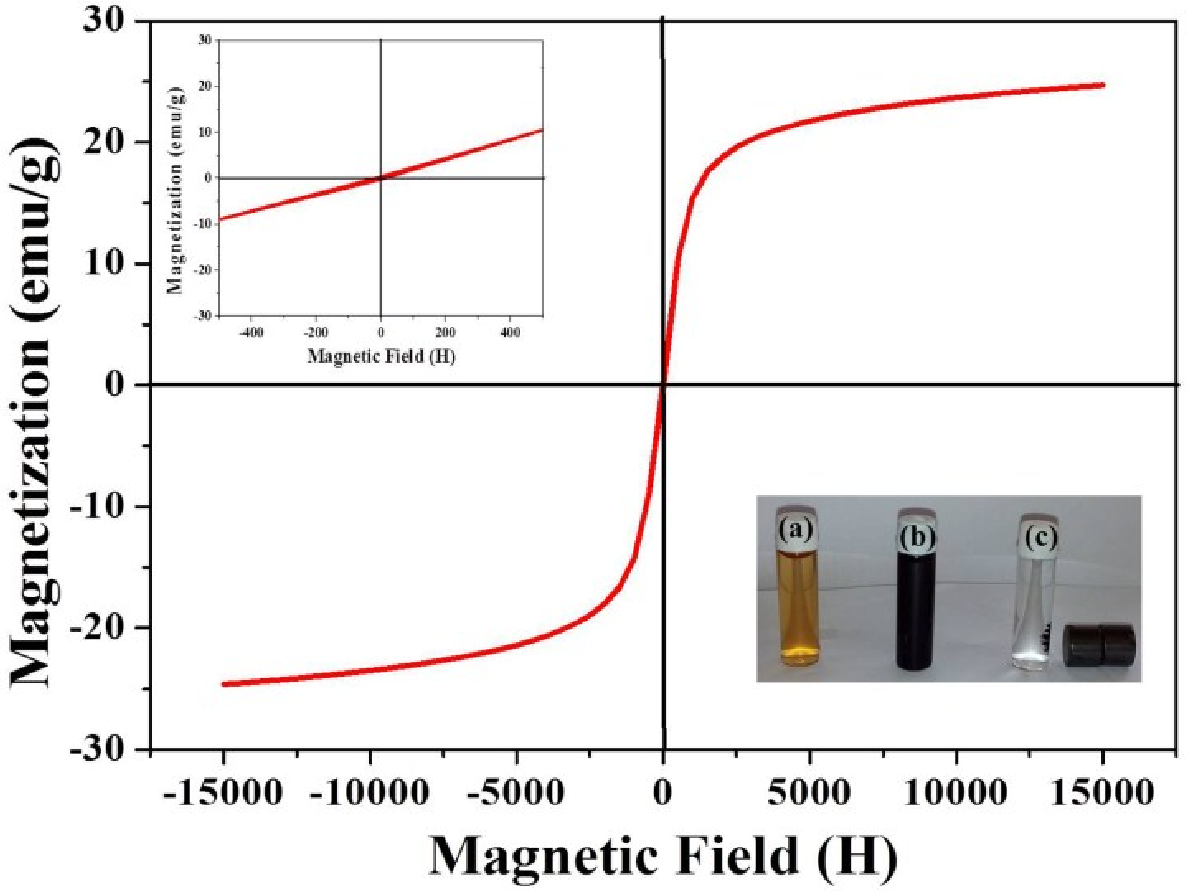

Fig.ESI5 The room temperature magnetization curve of Fe_3O_4@NCQDs NCs (top up left side inset show hysteresis loop in narrow expanded scale range of -0.5 kOe to 0.5 kOe and bottom right side inset show easy separation of the Fe_3O_4@NCQDs NCs using external magnet (a) NCQDs solution (b) colloidal suspension and (c) separation of synthesised Fe_3O_4@NCQs NCs in presence of external magnet)

Table ESI1: Isotherm parameters for MB dye solution onto Fe_3O_4@NCQDs NCs

Temp. (K)	Langmuir				Freundlich			Temkin		
	Q_o (mg/g)	b	R_L	R^2	K_F (mg/g)	n	R^2	A_T	b_T (J/mol)	R^2
293	24.888	2.679	0.436	0.985	19.491	1.838	0.987	27.252	450.957	0.977
298	24.480	2.157	0.428	0.989	17.069	1.842	0.993	22.265	469.384	0.974
303	24.414	1.834	0.414	0.946	16.317	1.811	0.997	19.357	477.986	0.944

Table ESI2 : Thermodynamic Parameters of MB dye adsorption onto Fe_3O_4@NCQDs nanocomposites

Temperature(K)	ΔG^0(KJ/mol)	ΔH^0(KJ/mol)	ΔS^0(KJ/mol)
293	-8.208		
298	-7.677	-38.591	-0.104
303	-7.203		

Table ESI3: Kinetic Parameters for adsorption of MB onto Fe3O4@NCQDs nanocomposites

Pseudo –first order			Pseudo-second order			
K_1(min^{-1})	Q_e(calc.)	R^2	K^2	Q_e(calc.)	h	R^2
0.228	3.125	0.939	0.194	14.14	38.91	0.999

Table ESI4: Comparison of adsorption capacity of nanocomposites for removal of methylene blue from aqueous solution

Nanocomposites	Maximum adsorption capacity q_m (mg g^{-1})	References
Poly(3,4-propylenedioxythiophene)/ MnO_2 composites	13.94	6
Magnetic graphene/calcium alginate (G-Fe_3O_4/CA)	17.27 at 20 °C	7
CS/Fe_3O_4/GO	30.01	8
OMWCNT-Fe_3O_4	41.50	9
Magnetic multi-wall carbon nanotube (Fe_3O_4-MWCNT)	15.74	10
Graphenenanosheet/magnetite (GNS/Fe3O4) composite	43.82	11
Fe3O4@NCQDs	24.5 at 20 ^{0}C	This work

Adsorption mechanism of dye on to the surface of Fe_3O_4@NCQDs NCs is based on electrostatic interaction between negatively charged Fe_3O_4@NCQDs NCs surfaces and cationic dyes. In basic media, the carboxyl surface functional group of the NCs become negatively charged while MB dyes become positively charged on nitrogen in the structure. These oppositely charged species attract each other through electrostatic interaction and form coordinate covalent bond and hence MB dye adsorbed on to the surface of Fe_3O_4@NCQDs NCs. In addition to electrostatic interaction, MB dye may also entrapped in hole space available in collected nanocomposites.

Scheme 2 Possible adsorption mechanism for adsorption of MB dye on to Fe_3O_4@NCQDs NCs

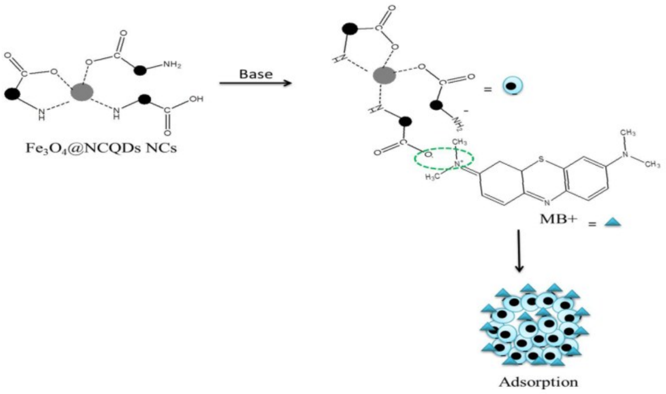

References

1. K. A. S. Fernando, S. Sahu, Y. Liu, W. K. Lewis, E. A. Guliants, A. Jafariyan, P. Wang, C. E. Bunker and Y.-P. Sun, ACS Appl. Mater. Interfaces, 2015, 7, 8363–8376.

2. S. N. Baker and G. A. Baker, Angew. Chem. Int. Ed Engl., 2010, 49, 6726–6744

3. E. Mansfield, in Modeling, Characterization, and Production of Nanomaterials, 2015, pp. 167–178.

4. M. Mahdavi, F. Namvar, M. Ahmad and R. Mohamad, Molecules, 2013, 18, 5954–5964.

5. K. Bhattacharya and P. Deb, Dalton Trans., 2015, 44, 9221–9229.

6. R. Jamal, L. Zhang, M. Wang, Q. Zhao and T. Abdiryim, *Materials International*. 26 (2016) 32–40.

7. N. Song, X.-L.Wu, S. Zhong, H. Lin and J.-R. Chen, *J. Mol. Liq.*212 (2015) 63–69.

8. H. V. Tran, L. T. Bui, T. T. Dinh, D. H. Le, C. D. Huynh and A. X. Trinh, *Materials Research Express*.4 (2017) 035701.

9. O. Duman, S. Tunç, T. G. Polat and B. K. Bozoğlan, *Carbohydr. Polym.*147 (2016) 79–88.

10. J.-L. Gong, B. Wang, G.-M.Zeng, C.-P.Yang, C.-G.Niu, Q.-Y.Niu, W.-J. Zhou and Y. Liang, *J. Hazard. Mater.164 (* 2009) 1517–1522.

11. L. Ai, C. Zhang and Z. Chen, *J. Hazard. Mater.*192 (2011) 1515–1524.
